# CD4^+^ T Cell-Receptor Repertoire Diversity is Compromised in the Spleen but Not in the Bone Marrow of Aged Mice Due to Private and Sporadic Clonal Expansions

**DOI:** 10.3389/fimmu.2013.00379

**Published:** 2013-11-19

**Authors:** Eric Shifrut, Kuti Baruch, Hilah Gal, Wilfred Ndifon, Aleksandra Deczkowska, Michal Schwartz, Nir Friedman

**Affiliations:** ^1^Department of Immunology, Weizmann Institute of Science, Rehovot, Israel; ^2^Department of Neurobiology, Weizmann Institute of Science, Rehovot, Israel

**Keywords:** TCR repertoire, aging, immune niche, clonal dominance, high throughput sequencing, TCR-seq, CD4+ T cells

## Abstract

Reduction in T cell receptor (TCR) diversity in old age is considered as a major cause for immune complications in the elderly population. Here, we explored the consequences of aging on the TCR repertoire in mice using high-throughput sequencing (TCR-seq). We mapped the TCRβ repertoire of CD4+ T cells isolated from bone marrow (BM) and spleen of young and old mice. We found that TCRβ diversity is reduced in spleens of aged mice but not in their BM. Splenic CD4+ T cells were also skewed toward an effector memory phenotype in old mice, while BM cells preserved their memory phenotype with age. Analysis of Vβ and Jβ gene usage across samples, as well as comparison of CDR3 length distributions, showed no significant age dependent changes. However, comparison of the frequencies of amino-acid (AA) TCRβ sequences between samples revealed repertoire changes that occurred at a more refined scale. The BM-derived TCRβ repertoire was found to be similar among individual mice regardless of their age. In contrast, the splenic repertoire of old mice was not similar to those of young mice, but showed an increased similarity with the BM repertoire. Each old-mouse had a private set of expanded TCRβ sequences. Interestingly, a fraction of these sequences was found also in the BM of the same individual, sharing the same nucleotide sequence. Together, these findings show that the composition and phenotype of the CD4+ T cell BM repertoire are relatively stable with age, while diversity of the splenic repertoire is severely reduced. This reduction is caused by idiosyncratic expansions of tens to hundreds of T cell clonotypes, which dominate the repertoire of each individual. We suggest that these private and abundant clonotypes are generated by sporadic clonal expansions, some of which correspond to pre-existing BM clonotypes. These organ- and age-specific changes of the TCRβ repertoire have implications for understanding and manipulating age-associated immune decline.

## Introduction

Effective T cell immunity is founded on a diverse T cell-receptor (TCR) repertoire. This diversity, generated by the V(D)J recombination mechanism in the thymus (1), is essential for coping with the plethora of invading and fast evolving pathogens. Loss of diversity, whether naturally occurring with age (2) or induced (3), is associated with increased susceptibility to infections, as well as reduced responses to vaccination (4, 5). One of the most dramatic manifestations of aging on the immune system is thymus involution. Toward old age, both in human and mice (6), the thymic epithelial tissue is replaced by connective and adipose tissue (7), causing reduction in *de novo* production of naive T cells through differentiation of precursor cells. Without thymic activity, naïve T cells are thought to be generated only through homeostatic proliferation of existing single-positive T cells (CD4^+^ and CD8^+^ T cells). In adult humans, this is the main mechanism for maintenance of the naïve T cell pool, while in mice there is evidence of lingering thymic output of naïve T cells (8).

Although both the phenotypic balance between memory and naïve T cells as well as the ratio of CD4^+^ to CD8^+^ T cells do not alter drastically with age (9), this does not indicate that the TCR repertoire is static (10). By using spectratyping to measure the distributions of TCR lengths across Vβ chains, studies have shown that both the CD8^+^ and CD4^+^ TCR repertoires in old mice were skewed compared to young mice (11). Moreover, the perturbations in CDR3 lengths were idiosyncratic to each individual. Deviations from the normal distribution for CDR3 lengths are assumed to be caused by massive T cell expansions during aging both in mice and humans (12). These changes in the composition of the TCR repertoire with aging can create vulnerability to pathogens, such as influenza (13), by providing incomplete clonal coverage.

The bone marrow (BM) is considered as the principal immune niche for both CD4^+^ and CD8^+^ memory T cells (14). Following massive clonal expansion during primary immune response against a pathogen, the contraction phase leaves only a small fraction of antigen-specific memory T cells. These long-lived cells reside mainly in the BM and represent the main T cell reservoir for secondary responses. In line with these observations, the BM was proposed as a “nest” for memory T cells (15), which can be expanded by homeostasis-driven proliferation for fighting viral infections (16), tumor (17), and even age-related cognitive loss (18, 19). It was demonstrated that antigen-specific CD4^+^ T lymphocytes which relocate throughout life to the BM have a slow turnover, but can fast react as professional memory CD4^+^ T cells, when stimulated (14).

Although effects of aging on TCR diversity have been evaluated in antigen-specific clones (10), it is still unclear what global changes the TCR repertoire undergoes during lifespan. These large-scale changes have been studied mostly using spectratyping (20, 21), a technique that maps the repertoire with very low resolution. Furthermore, the differences between immune niches, such as the BM and spleen (SPL), in terms of their distinct TCR repertoires and their development with age remain to be explored.

Here we show that the TCRβ repertoire of CD4^+^ T cells is shaped both by their immunological niche and by age. We used high throughput sequencing to map the murine TCRβ repertoire, of both splenic and BM-derived CD4^+^ T cells. Aged mice display a marked reduction in diversity of splenic T cells, while diversity of BM-derived T cells is relatively constant with age. Moreover, the TCRβ repertoire of splenic T cells in aged mice becomes more similar to the repertoire of BM-derived T cells. The loss of diversity in old mice is associated with expansion of tens to hundreds T cell clones, and occurs in parallel to segregation of the repertoires of different mice, creating distinct and private immune signatures in each aged individual. Finally, we evaluated clonal expansion and convergent recombination (22) in aging in order to find evidence for the mechanism that creates private repertoires in old age. We show multiple occurrences of sharing at the nucleotide (nt) level between TCR sequences derived from BM T cells and from massively expanded SPL T cell clones of the same aged animal. These results suggest that the degenerate repertoire in old age is shaped by rare events of massive clonal expansions which allow distinctive T cell clones to dominate the immune repertoire of individuals.

## Materials and Methods

### Animals

Inbred male 6- to 8-week-old C57BL/6 mice were supplied by the Animal Breeding Center of The Weizmann Institute of Science. Inbred male 17- to 20-months-old C57BL/6 mice were supplied by the National Institute on Aging (NIA). Aged mice were allowed 1 month adaptation period following shipment from the NIA to our laboratory. All animals were handled according to regulations formulated by The Weizmann Institute’s Animal Care and Use Committee and maintained in a pathogen-free environment.

### Sample preparation and CD4+ T cells isolation

Prior to tissue collection, mice were intracardially perfused with PBS. Spleens were mashed with a syringe plunger and treated with ammonium-chloride potassium (ACK) lysing buffer to remove erythrocytes. BM was extracted from the femur and tibiae of the mice. Single-cell suspensions of the samples were loaded on MACS column (Miltenyi Biotec) and CD4^+^ T cells were isolated according to manufacturer’s protocol.

### Flow cytometry and analysis

The following fluorochrome-labeled mAbs were used according to the manufacturers’ protocols: PercpCy5.5-conjugated anti-TCRβ, PE-conjugated anti-CD4, FITC-conjugated anti-CD44, and APC-conjugated anti-CD62L (BD Pharmingen and eBioscience). Cells were analyzed on an LSRII cytometer (BD Biosciences) using FACSDiva (BD Biosciences) and FlowJo (Tree Star) softwares. In each experiment, relevant negative-control groups and single-stained samples for each tissue were used to identify the populations of interest and to exclude others.

### Library preparation for TCR-seq

All libraries in this work were prepared and pre-processed as published (23). Briefly, we extracted total RNA from CD4^+^ T cells (from spleen or BM) of C57BL/6 mice using RNeasy Mini Kit (Qiagen). The RNA was reverse transcribed using SuperScript II reverse transcriptase (Invitrogen) and a TCR Cβ-specific primer linked to the 3′-end Illumina sequencing adapter. The resulting cDNA was then amplified using PCR (Phusion; Finnzymes) with a Cβ-3′adp primer and a set of 23 Vβ-specific 5′ primers, each of which was anchored to a restriction site sequence for the ACUI restriction enzyme. PCR products were then cleaned using QIAquick PCR purification kit (Qiagen), followed by enzymatic digestion with ACUI (New England BioLabs). The ACUI enzyme was used to cleave the amplicons such that sequencing starts closer to the V-D junction region. This allows for good coverage of CDR3β with a single Illumina read. This was followed by ligation of a 5′ Illumina adaptor (T4 ligase; Fermentas), which also contained a 3-nt tag for sample multiplexing. A second round of PCR amplification was performed, using primers for the 5′ and 3′ Illumina adapters. Final PCR products were run on a 2% agarose gel, cut at the desired length, and purified using Wizard SV Gel and PCR Clean-Up System (Promega) to produce the final library. The libraries were sequenced using Genome Analyzer II (Illumina).

### Pre-processing and error correction for raw reads

We filtered out raw reads containing bases with *Q*-value ≤30, and then separated the remaining reads according to their barcodes. Then, we aligned the reads to each of the germline Vβ/Jβ gene segments from IMGT (24) using the Smith–Waterman algorithm. Each read was assigned its best-aligning Vβ/Jβ if the number of matching nt (alignment length) was above a threshold: 11 nt for Vβ, 9 nt for Jβ. To reduce the effect of sequencing errors, we clustered (hierarchically) reads assigned the same Vβ and Jβ genes to correct up to 2 nt misincorporation errors. Then, we annotated the sequences by matching the Dβ to the junction, identifying deleted/inserted nt and elongated the read to its full CDR3β length (by IMGT convention). Finally, we translated the nt sequences into amino acid (AA) CDR3β. For the entire analysis here, we used only sequences that are fully annotated (V, J segments assigned), are in-frame (i.e., they encode for a functional peptide, without stop codons), have a cluster size of at least two and have less than 2 bp enzyme cleavage error. We also corrected the copy-number, to adjust for PCR and sub-sampling bias, as published (23).

### Statistical analysis

All statistical analysis was performed using R Statistical Software (R (25)). We also used ShortRead package (26) for the pre-processing pipeline, “ineq” package (27) to calculate the Gini coefficient and “ggplot2” (28) for generating figures. Statistical tests performed are stated in the text.

## Results

### Diversity of the splenic TCRβ repertoire is compromised in old mice

We aimed to explore the changes in the repertoire landscape at old age, with emphasis on evaluating the diversity of the TCR repertoire. To accomplish this, we measured using TCR-seq the TCRβ repertoire in mice from two age groups: 6–8 weeks old (termed “young,” *n* = 3) and 17–20 months old (“old,” *n* = 3). In addition, to evaluate the differences between immune organs, we isolated CD4^+^ T cells from the SPL and BM of each mouse. Properties of all samples are detailed in Table 1. On average, we have ∼2e6 sequence reads that have passed the quality threshold (see Materials and Methods), for each sample. These quality-filtered reads produced an average of ∼2.8e5 reads that could be annotated with full CDR3β sequence properties (see Materials and Methods), including translation to an in-frame AA sequence. BM samples resulted in about 10-fold less annotated reads compared with SPL samples. In total, we have found 108,124 distinct CDR3β AA sequences in these 12 samples.

**Table 1:** **Sample properties**.

Sample name	Age group	Organ	Raw reads	Total annotated	Unique reads
ySP1	Young	Spleen	2,653,822	1,075,670	77,991
ySP2	Young	Spleen	804,529	134,372	12,298
ySP3	Young	Spleen	1,426,160	470,363	32,293
yBM1	Young	BM	1,538,612	84,111	6,167
yBM2	Young	BM	1,211,410	4,492	722
yBM3	Young	BM	2,857,600	65,879	6,183
oSP1	Old	Spleen	4,325,524	459,288	18,363
oSP2	Old	Spleen	2,790,495	384,664	17,004
oSP3	Old	Spleen	2,226,719	579,364	9,594
oBM1	Old	BM	1,747,231	16,653	1,443
oBM2	Old	BM	2,036,502	87,758	8,964
oBM3	Old	BM	1,533,444	52,905	4,076

**Figure 1:**
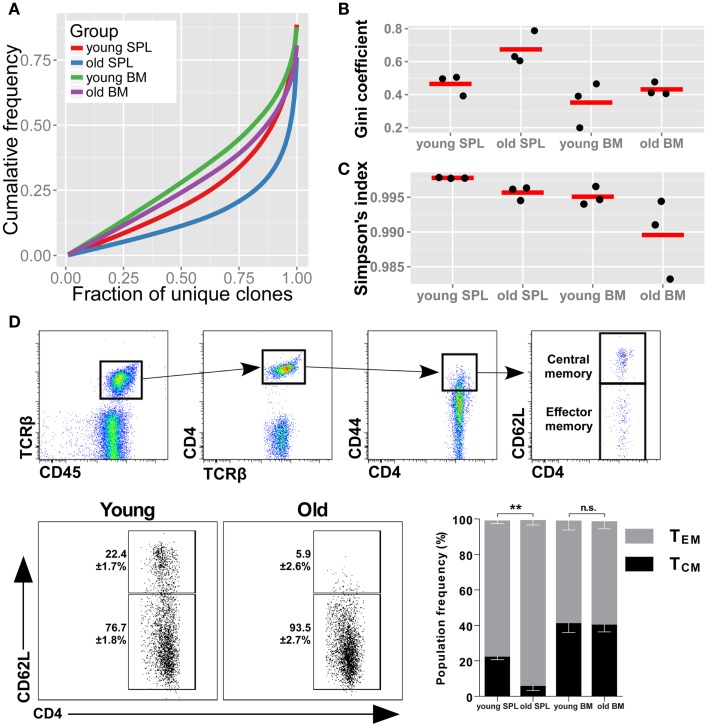
**TCRβ repertoire is less diverse in the spleens of old mice**. **(A)** Skewness of the TCRβ repertoire for CD4+ T cells from spleen and BM of young and aged mice. For each mouse, clonotypes were ordered by frequency. We then compared the cumulative frequency at each rank (normalized to sample size). From the curves, which represent the mean for each group, we observe that old SPL has the most skewed repertoire. **(B)** Gini coefficient per group. Horizontal lines represent the mean for each group and dots are individual samples. Old SPL group has the highest values, thus it is the most skewed. **(C)** Simpson’s diversity index calculated for each mouse (dots). Horizontal lines represent the mean for each group. The old SPL group has a significantly lower diversity than the young SPL group. **(D)** Phenotypic changes of CD4+ memory T cells in aging. Top panels show flow cytometric gating strategy. Old spleen samples have significantly higher percentage of effector memory T cells compared to young spleen samples (bottom panels and bar graph). BM samples have similar central/effector memory ratios across age groups (mean ± SE of each group (*n* = 4–5 per group; **P < 0.01; Student’s *t* test).

To evaluate the diversity of TCR sequences in the samples, we first checked the cumulative frequencies of clonotypes, ordered by their rank. Hence, we sorted all of the AA clonotypes by their frequency in ascending order. To adjust for varying sample size, we normalized the rank between 0 (the rarest clone) to 1 (the most abundant clone). We then calculated the mean cumulative frequency for increasing rank bins across mice belonging to the same group (Figure 1A). In this representation, also known as a Lorenz curve, a repertoire that has maximal diversity (i.e., all clonotypes are present in equal frequency), will be plotted as a straight line across the diagonal. In contrast, a skewed repertoire (i.e., few and very abundant clonotypes dominate the sample) will deviate below the diagonal with a sharp incline only toward the higher ranks. We observe that the most skewed repertoire belongs to the old SPL, while BM from young mice is the most diverse of the repertoires studied here. The old SPL repertoire had a decreased diversity compared with that of the young SPL, while the BM repertoire showed only a slight decrease in diversity with age.

As another measure for repertoire skewness, we applied the Gini coefficient for inequality, used to measure evenness of wealth distribution in economics, which was applied recently for evaluation of TCR repertoire diversity (29). High values of the Gini coefficient, which ranges from 0 to 1, are indicative of a skewed repertoire. We calculated the Gini coefficient for each of the samples and grouped the results by organ and age (Figure 1B). The Gini coefficient is highest for the old SPL group, consistent with the Lorenz curve. The decline in clonal equality with age is evident in the spleen (*p* < 0.05, Student’s *t* test), but not in the BM (*p* = 0.42).

We used an additional metric for measuring diversity in TCR samples, the Simpson’s diversity index (30, 31). This metric takes into account both the number of unique clonotypes and their relative frequency. The Simpson’s diversity index represents the probability that any two clonotypes randomly drawn from the sample will have different sequences. The Simpson index ranges from 0 to 1, with 1 representing maximal diversity, i.e., all clonotypes are present in equal sizes. For each sample, we calculated the mean Simpson’s diversity index for 500 randomly sampled clones in 1,000 iterations (Figure 1C). Consistent with our observation for the skewness of the repertoire in old mice, the old SPL group has a significantly lower diversity compared with the young SPL [*p* < 0.001, permutations test, see Ref. (30)]. To conclude, we find that the diversity of the splenic TCRβ repertoire is significantly reduced at old age, based on the three analysis methods. Reduction in the diversity of the BM repertoire is minimal and not statistically significant with current sample sizes.

### Splenic CD4^+^ T cells are skewed toward an effector memory phenotype

Next, we wished to determine whether these two immunological compartments, SPL and BM, age differently in terms of the memory phenotype of CD4^+^ T cells. Using flow cytometry, we measured the proportions of effector memory (T_EM_) and central memory (T_CM_) phenotypes in the CD4^+^ memory T cell compartment (Figure 1D). We found that T_EM_ CD4^+^ T cells are significantly more abundant in spleens of old mice (93.5 ± 2.7%) compared to young mice (76.7 ± 1.8%). Increase in the effector phenotype could point to expansion driven by antigen-specific responses and suggests that these CD4^+^ T_EM_ cells contribute to the skewed repertoire we observe in old spleen. No significant change in T_EM_/T_CM_ balance was observed in BM samples between age groups (Figure 1D), suggesting maintenance of the phenotypic balance in the BM niche.

### Organ specific patterns of Vβ and Jβ segment usage are constant with age

**Figure 2:**
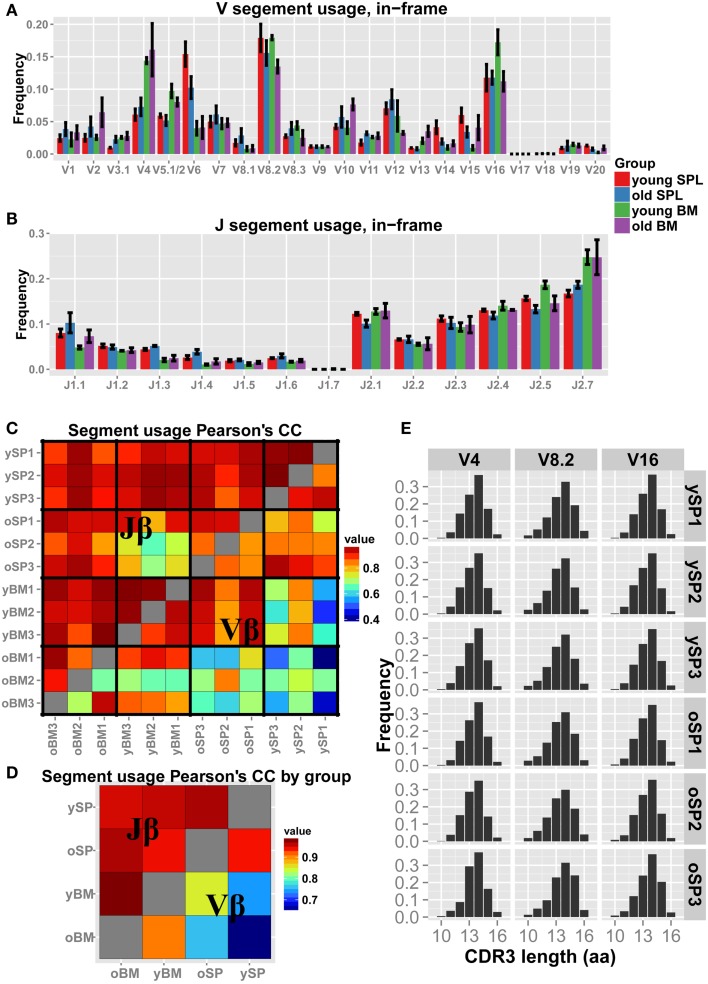
**Vβ and Jβ usage in aged mice**. **(A,B)** Each bar represents the mean frequency of a gene segment in that group of mice. Error bars are SEM. **(C)** Pairwise correlations of Vβ and Jβ usage between all pairs of mice. We observe higher correlations between mice in Jβ usage (upper triangle) compared to Vβ usage (lower triangle). **(D)** Correlation of the gene segment usage between all pairs of mice averaged over groups. We detect a general high correlation in the data, with inter-tissue similarity across age groups. BM = bone marrow, SPL = spleen, *n* = 3 for all groups. **(E)** “Virtual” spectratypes. Each bar represents the relative frequency of a particular CDR3 length (in amino acids) for three representative Vβ segments, stated above each panel, measured across individual SPL samples. No significant changes could be detected in CDR3 length distributions across age groups.

We next examined how the gene segment usage changes with aging (Figures 2A,B). Qualitatively, the Jβ distributions look similar in all samples, with few segments that differ in frequency between spleen and BM. For example, Jβ1.3 is over-expressed in SPL samples (both young and old) compared to BM samples, whereas Jβ2.7 is under-expressed in SPL samples. The Vβ distributions vary between samples to a larger extent, evidenced also by the overall lower correlation scores (Figure 2C). Inter-group correlations in gene segment usage (Figure 2D) show similarity between organ specific repertoires across age, which is higher than between repertoires of different organs within age groups. Thus, gene segment usage is more similar between young and old BM samples, and between young and old SPL samples; it is less similar between BM and SPL samples, both in young and aged mice. This suggests that the tissue microenvironment plays a major factor in shaping of the TCRβ repertoire.

Spectratyping analysis is often used to test for skewness and clonal dominance in TCR repertoires. Thus, we generated virtual spectratypes of CDR3 length distributions for sequences grouped by their Vβ segment. Specifically, as our analysis detected skewness in old SPL samples, we aimed to test if a particular CDR3 length was dominant in that age group (see Figure 2E for representative plots). This analysis revealed length distributions that are largely homogenous across samples, with neither significant changes in skewness nor enrichment of a specific CDR3 length. Thus, coarse analysis of the CD4^+^ TCR repertoire, comparing gene segment usage as well as spectratyping analysis, could not explain the measured loss in diversity in aged mice.

### BM TCR repertoires are similar between mice and age groups, while SPL TCR repertoires change and become private with age

**Figure 3:**
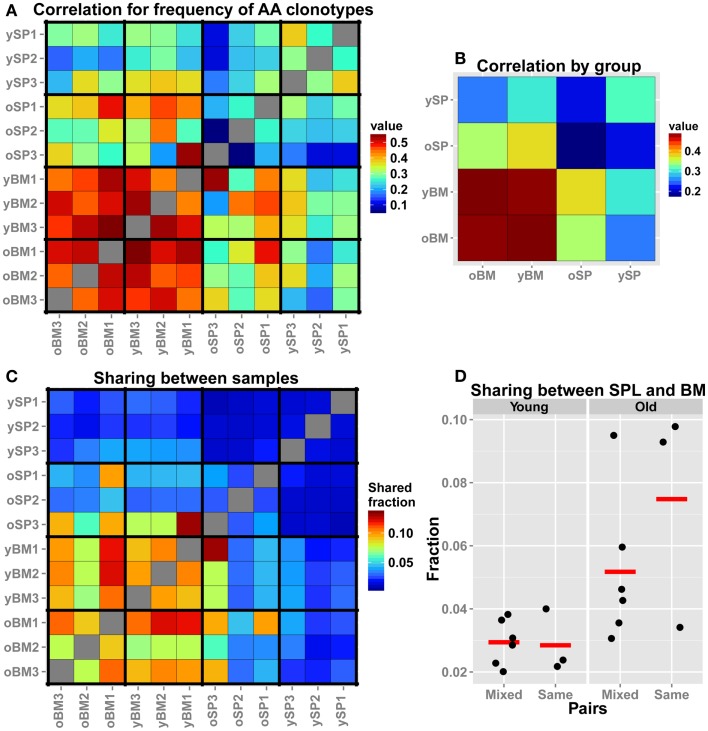
**Comparison of the TCR repertoires in different tissues and age groups at the level of AA clonotypes**. **(A)** For each pair of mice, we calculated the Spearman’s correlation for frequencies of clonotypes that are shared by that pair. As the frequency distribution of TCR repertoire is over-dispersed, we used the log-transformed values as input for the rank correlation test. The BM groups have high similarity between the samples, and also between age groups. **(B)** For each group of mice (*n* = 3), we averaged the correlation scores of **(A)**. Scores along the diagonal indicate the intra-group similarity. Again, the homogeneity in the BM samples within groups and across age is evident. In addition, the repertoire of the old SPL group has a higher correlation with the young BM and old BM groups, and a low intra-group correlation. **(C)** Sharing of clones between samples. From each sample, we randomly chose 300 AA clones and calculated pairwise sharing. The values represent the fraction of the sample that is shared between any particular pair, averaged over 100 iterations. BM repertoires show high level of sharing between individual mice and across age groups. **(D)** A comparison of sharing between BM and SPL repertoires within the same animal or from different animals (Mixed).

TCR-seq allows for comparison of repertoires at a higher resolution, beyond gene segment usage or CDR3 length distributions, by analysis at the level of individual TCR sequences. Hence, we assessed similarity between samples by comparing frequencies of overlapping clonotypes. This comparison is more stringent than measuring Vβ/Jβ usage, as we search for the same exact AA clonotypes and compare their observed frequency in each pair of samples. We find that, in accordance to our findings for the Vβ/Jβ usage, all BM samples, regardless of age, display a high similarity in the frequencies of shared AA clonotypes (Figure 3A). We also notice that the old SPL group is non-homogenous, i.e., the inter-sample similarity (between individuals) is lower when compared to that found in the young SPL group. To further illustrate this point, we calculated the mean of all pairwise correlation coefficients for each group comparison (e.g., young SPL vs. old SPL, young SPL vs. old BM, etc.). This analysis estimates the overall similarity between groups (off-diagonal elements in the matrix of Figure 3B) and also between samples from the same group (diagonal elements, Figure 3B). We observe that the old SPL group has the lowest within-group correlation, and that it is similar to some degree to the young BM and old BM groups, but not to the young SPL group. This suggests that, in aged mice, some clones that were resident in the BM at younger age have migrated to the spleen. BM samples are similar both between and within age groups (Figure 3B).

As another measure for similarity, we evaluated the number of overlapping sequences between pairs of samples. In order to control for different sample sizes (Table 1), we randomly sampled a collection of 300 clonotypes from each sample and tested how many of these clonotypes are shared between all possible pairs of samples. We iterated this test 100 times to reduce sampling noise and calculated the mean for each pair of samples (Figure 3C). We found that the fraction of shared clones within the BM samples is higher than within SPL samples of both age groups. Average sharing of 10% was found between the young BM samples and 9% between the old BM samples. Sharing between SPL samples was much lower (1% for young SPL samples and 3% for old SPL samples). Moreover, 10% of clones are shared on average between young and old BM samples, whereas only 1% are shared between young and old SPL samples. This supports our previous results showing that aging of the immune system affects the composition of the SPL repertoire, but has little influence on the repertoire of BM-resident CD4^+^ T cells.

We next focused on the inter-tissue sharing of clones by comparing the overlap between SPL and BM repertoires from the same animal, to the overlap observed between SPL and BM repertoires taken from different animals (Figure 3D). In general, there are more clones shared across niches in old animals compared to young animals. Interestingly, we notice that in two out of three old mice, more clones are shared between SPL and BM repertoires that are derived from the same animal, compared with SPL and BM repertoires that are derived from different animals. However, we do not observe this pattern in young animals. These results suggest that with age, a private set of clones is expanded in each individual, contributing to the reduction in SPL repertoire diversity. Furthermore, as the overlap between the repertoires of the spleen and BM niches increases in old age, the repertoire of the whole animal becomes less diverse and degenerate.

### Clonal dominance is prevalent in splenic CD4^+^ T cells from old mice

**Figure 4:**
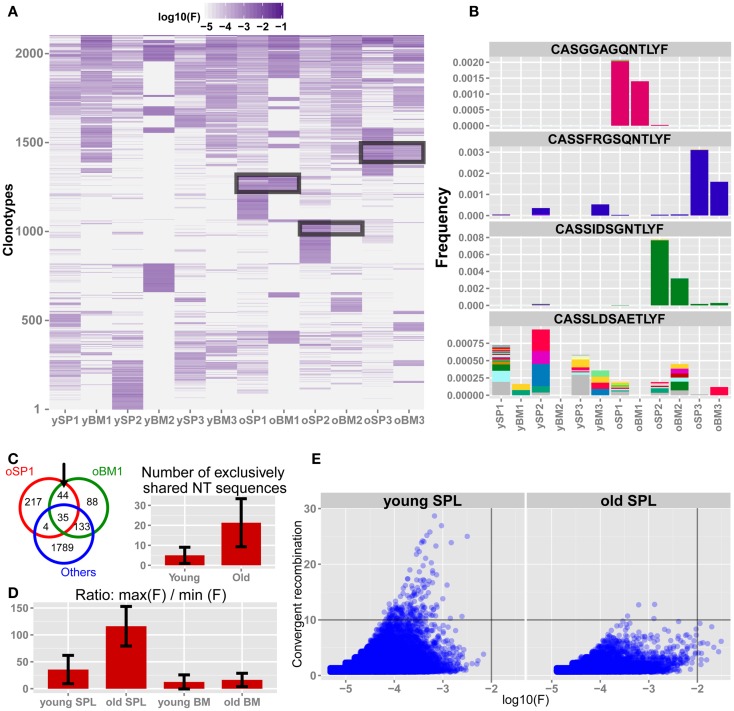
**The TCRβ repertoire of old mice is shaped by private clonal expansions**. **(A)** Top 300 ranking AA clonotypes from all samples (total of 2,108 clonotypes) were clustered using Euclidian distance. Paired samples from the same animal are adjacent to each other. In old SPL there are subsets of enriched clones, which are unique to each mouse. Also, part of that subset is present in high frequency in the matching BM sample from the same animal (black frames). **(B)** Frequencies of four selected AA clonotypes across all samples. Stacked bars show the nt sequences, uniquely colored, that encode the AA sequence stated above the panel. Top 3 panels show representative clonotypes that are expanded and private in SPL and BM of old mice. Remarkably, these AA sequences are encoded by the same nt sequence in the spleen and BM of these animals. The bottom panel shows a typical abundant AA clonotype in young SPL samples, showing a high level of convergent recombination across most samples in which it is found. **(C)** Sharing of top 300 nt sequences between SPL and BM of the same animal. For each mouse, we calculated the number of exclusively shared nt sequences. The Venn diagram (left) shows an example in which 44 sequences are exclusively shared by BM and SPL of old mouse #1, and are not found in any other sample. Bar plot (right) shows that there are on average more exclusively shared nt sequences within SPL and BM of the same animal in old mice compared to young mice. Error bars indicate standard error. **(D)** Evaluation of clonal dominance. Bars show the average ratio between the frequency of the most frequent and the least frequent nt sequence encoding for the same AA sequence. Values were calculated for the top 300 AA clonotypes from each sample. The ratio is significantly higher in old SPL samples compared to young SPL (*p* < 0.05, Student’s *t* test), suggesting clonal dominance. **(E)** Convergent recombination (# of nt sequences encoding each AA sequence) of AA clonotypes is plotted against their frequency. In old SPL the scatter shows a subset of high frequency clones that are encoded by less than 10 nt sequences (lower-right quadrant). In contrast, in young SPL many clonotypes show high convergent recombination (upper-left quadrant).

Following our observation that the repertoire of splenic T cells from old mice becomes less diverse, private and more similar to the BM repertoire, we next focused on analysis of properties of specific clones that contribute to these phenomena. To that end, we first pooled the top 300 AA clonotypes from each sample to a unified list of 2,108 unique AA sequences. Then, we clustered the log-transformed frequencies for all the sequences in the unified list across all samples (Figure 4A). We observe that young SPL samples share many of these top clonotypes, indicating a baseline similarity in the repertoire of young mice. In contrast, the old SPL samples are distinct from each other and each individual presents a unique subset of highly abundant clonotypes, which have intermediate to low frequencies in the young SPL. Furthermore, BM samples from old mice share several abundant clones with their paired SPL samples, consistent with the intra-mouse sharing we observed above (Figure 3D).

To reveal if the sharing of AA clonotypes in the old mice samples is also present at the nt level, we picked three representative AA clonotypes that are shared between the SPL and BM samples of old mice (Figure 4B). Strikingly, we found that in all three cases the same nt sequence encodes the AA clonotype that is highly frequent in both the SPL and BM. This is a strong indication that the event of clonal expansion occurred for a particular T cell clone, causing clonal dominance in aged mice, which is evident in both BM and SPL of the animal. In contrast, AA clonotypes that are highly frequent in young SPL samples typically show high convergent recombination where many nt sequences encode for the same AA clonotype (Figure 4B).

Following this observation, we extended this analysis and counted the number of nt clones that are shared between BM and SPL of the same animal, which are not shared by any other sample (SPL or BM) from other animals. We find that more nt sequences are shared exclusively between the BM and SPL of the same animal in old mice, but not in young mice **(**Figure 4C**)**. This reflects again the private repertoires generated in old age, in parallel with the increased similarity between the BM and SPL repertoires.

These results suggest that expanded clonotypes in aged mice show clonal dominance, that is, the same TCR nt sequence is responsible for most observed TCRs that have the same AA sequence. To test this hypothesis, we directly calculated clonal dominance in old mice compared to young mice. For each mouse, we considered only the 300 most abundant AA clonotypes that are encoded by at least two distinct nt sequences. Then, we calculated the ratio between frequencies of the most abundant nt sequence and the least abundant nt sequence encoding each AA clonotype (Figure 4D). In the old SPL group, there is over a 100-fold difference on average, between the maximal frequency and the minimal frequency of nt sequences coding for the same AA sequence. This ratio, *R*, is significantly higher in old SPL samples (*R* = 116) than in young SPL (*R* = 36). The ratio in the BM is low for both age groups (*R* = 13 for young BM and *R* = 16 for old BM). This supports the hypothesis that expanded clonotypes in the old SPL represent events of massive clonal expansion of a particular T cell clone.

Finally, to illustrate the global changes that the repertoire undergoes with aging, we plot the number of unique nt sequences encoding for the same AA clonotype (convergent recombination level) against the frequency of that clonotype, for all AA clonotypes from all spleen samples (Figure 4E). We notice that the old SPL group contains a subset of clonotypes that are highly expanded and have low to moderate convergent recombination (encoded by up to 10 different nt sequences). Clonotypes with similar properties are not found in the young SPL group. This supports the hypothesis that sporadic clonal expansion is a major factor in shaping the repertoire in old mice. Also, there are very few clonotypes (*n* = 3, 0.007% of the clonotypes) with convergent recombination higher than 10 in the old SPL group, whereas in the young SPL group there are many such clonotypes (*n* = 152, 0.16%).

## Discussion

The immune system undergoes changes with aging, contributing to an overall increase in neurodegenerative diseases and decrease in autoimmune inflammatory diseases. In addition, susceptibility to infectious diseases inclines with age (32) due to a combination of several factors such as immune senescence (33), transcriptional changes (34), and loss of *de novo* production of naïve T cells (35). Aged individuals are particularly vulnerable to newly encountered pathogens, as TCR diversity is severely diminished in old age. Here we explored the consequences of aging on the TCR repertoire in mice, with focus on the underlying causes for loss of diversity.

We applied TCR-seq on CD4^+^ T cells isolated from the BM and spleen of young and aged mice. First, our focus was on measuring diversity across all samples. As observed before for CD8^+^ T cells (20), we found that the diversity of the splenic CD4^+^ T cell compartment also declines in aged mice. Reduced diversity was revealed both by high Gini inequality coefficient and a low Simpson diversity index. However, in BM samples only a minor reduction of diversity was detected with aging, which was not statistically significant. Examining the memory phenotype of the CD4^+^ T cells in these two niches revealed a similar pattern; while in the spleen the memory phenotype of the cells strongly shifted toward effector memory during aging, the proportions of effector and central memory T cells in the BM remained constant. This can be attributed to the nature of the BM immune niche as an immune privileged hematopoiesis site (36), allowing maintenance of only a small subset of T cell clonotypes thus less affected by clonal attrition. Clonal expansion in the BM may be inhibited due to the abundance of quiescence-inducing signals which prevent extensive proliferation of stem/progenitor cells, found in the BM as a hematopoietic niche (37).

We next evaluated the patterns of gene segment usage in our dataset. Consistent with our previous observation (23), Jβ usage is similar across samples and is not influenced significantly by tissue specificity or age. In general, the highest correlation in Vβ and Jβ usage is observed between the BM samples from both age groups, indicating a relatively stable TCR repertoire in the BM niche across age. However, analysis of distributions of gene segment usage and of CDR3 length do not show significant age-related differences that can explain the observed loss of splenic repertoire diversity. Thus, certain aspects of repertoire dynamics can be evaluated only with increased resolution, achieved by high throughput methods such as TCR-seq. This may have masked clear detection of decline in repertoire diversity with age in the CD4^+^ compartment in previous studies that used spectratyping for evaluation of repertoire diversity (21, 38).

Gene segment usage only partially depicts the set of specificities encompassed by the TCR repertoire, thus we focused on the deepest functional level of the repertoire, the CDR3 AA sequence. Here, the similarity between the repertoires of BM niches, even between young and old mice, is emphasized. AA clonotypes in the BM are shared and their frequencies are well-correlated across the samples from different individuals, and also across age groups. This supports the notion of a relatively static composition of clonotypes in the BM niche which maintains the structure of the TCR repertoire of BM-resident CD4^+^ T cells. In contrast, the SPL samples from aged mice display very distinct repertoires, evidenced by a low intra-group correlation for frequencies of shared AA clonotypes and a low sharing of TCR sequences between mice of this group. This suggests that the loss of diversity we detect in old mice is manifested by private immune responses during lifetime, where in each individual a particular subset of TCR specificities is amplified to dominance. This is in agreement with the pattern observed in antigen-specific response in aged mice (10) but on a more global scale. We observe tens to hundreds of clones that are private and significantly expanded in each old individual’s spleen, indicating that decline in repertoire diversity is caused by expansion of a large number of clones through life. Moreover, the repertoire of the old spleen becomes more similar to that of the BM in the aged mice. Of note, in two out of three aged mice, more clonotypes are shared between BM and SPL niches of the same animal compared with sharing between different animals. This trend of exclusive sharing between BM and SPL niche of the same animal is not evident in any of the three young mice. Together with the larger similarity between repertoires of the SPL niche in aged mice to that of the BM, this suggests that specific clones from the BM niche expanded significantly in the periphery, contributing to a skewed, degenerate, and private repertoire in the old SPL. Our phenotypic analysis (Figure 1D) suggests that these expanded clones acquire an effector memory phenotype in the old spleen, but more specific analysis is required to validate this hypothesis. In addition, extending our dataset to include additional mice could provide further evidence for the private repertoires in the aged spleen, and for increased similarity between BM and SPL repertoires that we observe in aged mice.

Lastly, we focus on those clones that are common to SPL and BM tissues of aged mice, but exclusive to each animal. We detect clonal dominance in these expanded groups of cells, with the same nt sequence present in high frequency in both niches. The chance of this expansion to occur in two independent events of clonal expansion is highly unlikely. As we find the same exact nt sequence in the BM and SPL of the same animal, we propose that sporadic clonal expansion is the mechanism that shapes the TCR repertoire in aging. This clonal dominance can be realized in the aging immune system, as the “void” created by clonal senescence and exhaustion (39) is more easily filled with rapidly dividing T cell clones. A similar phenomenon was described in other models of similar low grade, chronic sterile innate inflammation, such as obesity, where TCR repertoire is restricted (40). The mechanisms that generate these rare expansions can be response to self-antigens (18), latent infections (32), or driven by accumulating mutations (41).

In summary, we showed that diversity of the splenic CD4^+^ TCR repertoire declines with age, while the BM repertoire remains largely unchanged. Our results suggest that with age, the TCRβ repertoire of each individual focuses on a certain subset of few hundreds clones out of the potential repertoire, and there is large variability between the subset each individual maintains. This attrition can be explained by a reduction in thymic output of naïve cells with age along with sporadic clonal expansion, which contribute to the clonal dominance we observe in old mice. As a consequence, this phenomenon should be considered when addressing vaccination of the elderly population.

## Conflict of Interest Statement

The authors declare that the research was conducted in the absence of any commercial or financial relationships that could be construed as a potential conflict of interest.

## References

[1] Bassing CH Swat W Alt FW The mechanism and regulation of chromosomal V(D)J recombination. Cell (2002) 109(Suppl):S45–55 10.1016/S0092-8674(02)00675-X 11983152

[2] Naylor K Li G Vallejo AN Lee W-W Koetz K Bryl E The influence of age on T cell generation and TCR diversity. J Immunol (2005) 174:7446–52 15905594 10.4049/jimmunol.174.11.7446

[3] Nanda NK Apple R Sercarz E Limitations in plasticity of the T-cell receptor repertoire. Proc Natl Acad Sci U S A (1991) 88:9503–7 10.1073/pnas.88.21.9503 1719532 PMC52746

[4] Kang I Hong MS Nolasco H Park SH Dan JM Choi J-Y Age-associated change in the frequency of memory CD4+ T cells impairs long term CD4+ T cell responses to influenza vaccine. J Immunol (2004) 173:673–81 15210831 10.4049/jimmunol.173.1.673

[5] Wu Y-CB Kipling D Dunn-Walters DK Age-related changes in human peripheral blood IGH repertoire following vaccination. Front Immunol (2012) 3:193 10.3389/fimmu.2012.00193 22787463 PMC3391689

[6] Linton PJ Dorshkind K Age-related changes in lymphocyte development and function. Nat Immunol (2004) 5:133–9 10.1038/ni1033 14749784

[7] Bains I Thiébaut R Yates AJ Callard R Quantifying thymic export: combining models of naive T cell proliferation and TCR excision circle dynamics gives an explicit measure of thymic output. J Immunol (2009) 183:4329–36 10.4049/jimmunol.0900743 19734223

[8] Den Braber I Mugwagwa T Vrisekoop N Westera L Mögling R de Boer AB Maintenance of peripheral naive T cells is sustained by thymus output in mice but not humans. Immunity (2012) 36:288–97 10.1016/j.immuni.2012.02.006 22365666

[9] Goronzy JJ Lee W-W Weyand CM Aging and T-cell diversity. Exp Gerontol (2007) 42:400–6 10.1016/j.exger.2006.11.016 17218073 PMC2680153

[10] Rudd BD Venturi V Li G Samadder P Ertelt JM Way SS Nonrandom attrition of the naive CD8+ T-cell pool with aging governed by T-cell receptor:pMHC interactions. Proc Natl Acad Sci U S A (2011) 108:13694–9 10.1073/pnas.1107594108 21813761 PMC3158207

[11] Mosley RL Koker MM Miller RA Idiosyncratic alterations of TCR size distributions affecting both CD4 and CD8 T cell subsets in aging mice. Cell Immunol (1998) 189:10–8 10.1006/cimm.1998.1369 9758689

[12] Schwab R Szabo P Manavalan JS Weksler ME Posnett DN Pannetier C Expanded CD4+ and CD8+ T cell clones in elderly humans. J Immunol (1997) 158:4493–9 9127016

[13] Yager EJ Ahmed M Lanzer K Randall TD Woodland DL Blackman MA Age-associated decline in T cell repertoire diversity leads to holes in the repertoire and impaired immunity to influenza virus. J Exp Med (2008) 205:711–23 10.1084/jem.20071140 18332179 PMC2275391

[14] Tokoyoda K Zehentmeier S Hegazy AN Albrecht I Grün JR Löhning M Professional memory CD4+ T lymphocytes preferentially reside and rest in the bone marrow. Immunity (2009) 30:721–30 10.1016/j.immuni.2009.03.015 19427242

[15] Di Rosa F Pabst R The bone marrow: a nest for migratory memory T cells. Trends Immunol (2005) 26:360–6 10.1016/j.it.2005.04.011 15978522

[16] Slifka MK Whitmire JK Ahmed R Bone marrow contains virus-specific cytotoxic T lymphocytes. Blood (1997) 90:2103–8 9292550

[17] Feuerer M Beckhove P Bai L Solomayer EF Bastert G Diel IJ Therapy of human tumors in NOD/SCID mice with patient-derived reactivated memory T cells from bone marrow. Nat Med (2001) 7:452–8 10.1038/86523 11283672

[18] Baruch K Ron-Harel N Gal H Deczkowska A Shifrut E Ndifon W CNS-specific immunity at the choroid plexus shifts toward destructive Th2 inflammation in brain aging. Proc Natl Acad Sci U S A (2013) 110:2264–9 10.1073/pnas.1211270110 23335631 PMC3568380

[19] Ron-Harel N Segev Y Lewitus GM Cardon M Ziv Y Netanely D Age-dependent spatial memory loss can be partially restored by immune activation. Rejuvenation Res (2008) 11:903–13 10.1089/rej.2008.0755 18803478

[20] Ahmed M Lanzer KG Yager EJ Adams PS Johnson LL Blackman MA Clonal expansions and loss of receptor diversity in the naive CD8 T cell repertoire of aged mice. J Immunol (2009) 182:784–92 19124721 10.4049/jimmunol.182.2.784 PMC2724652

[21] Callahan JE Kappler JW Marrack P Unexpected expansions of CD8-bearing cells in old mice. J Immunol (1993) 151:6657–69 8258683

[22] Quigley MF Greenaway HY Venturi V Lindsay R Quinn KM Seder RA Convergent recombination shapes the clonotypic landscape of the naive T-cell repertoire. Proc Natl Acad Sci U S A (2010) 107:19414–9 10.1073/pnas.1010586107 20974936 PMC2984183

[23] Ndifon W Gal H Shifrut E Aharoni R Yissachar N Waysbort N Chromatin conformation governs T-cell receptor Jβ gene segment usage. Proc Natl Acad Sci U S A (2012) 109:15865–70 10.1073/pnas.1203916109 22984176 PMC3465372

[24] Lefranc M-P Giudicelli V Ginestoux C Jabado-Michaloud J Folch G Bellahcene F IMGT, the international ImMunoGeneTics information system. Nucleic Acids Res (2009) 37:D1006–12 10.1093/nar/gkn838 18978023 PMC2686541

[25] Core Team R R: A Language and Environment for Statistical Computing. Vienna (2013).

[26] Morgan M Anders S Lawrence M Aboyoun P Pagès H Gentleman R ShortRead: a Bioconductor package for input, quality assessment and exploration of high-throughput sequence data. Bioinformatics (2009) 25:2607–8 10.1093/bioinformatics/btp450 19654119 PMC2752612

[27] Zeileis A Ineq: Measuring Inequality, Concentration, and Poverty. (2012).

[28] Wickham H Ggplot2: Elegant Graphics for Data Analysis. New York: Springer (2009).

[29] Thomas PG Handel A Doherty PC La Gruta NL Ecological analysis of antigen-specific CTL repertoires defines the relationship between naive and immune T-cell populations. Proc Natl Acad Sci U S A (2013) 110:1839–44 10.1073/pnas.1222149110 23319654 PMC3562793

[30] Venturi V Kedzierska K Turner SJ Doherty PC Davenport MP Methods for comparing the diversity of samples of the T cell receptor repertoire. J Immunol Methods (2007) 321:182–95 10.1016/j.jim.2007.01.019 17337271

[31] Mehr R Sternberg-Simon M Michaeli M Pickman Y Models and methods for analysis of lymphocyte repertoire generation, development, selection and evolution. Immunol Lett (2012) 148:11–22 10.1016/j.imlet.2012.08.002 22902400

[32] Nikolich-Zugich J Ageing and life-long maintenance of T-cell subsets in the face of latent persistent infections. Nat Rev Immunol (2008) 8:512–22 10.1038/nri2318 18469829 PMC5573867

[33] Goronzy JJ Weyand CM Understanding immunosenescence to improve responses to vaccines. Nat Immunol (2013) 14:428–36 10.1038/ni.2588 23598398 PMC4183346

[34] Chen G Lustig A Weng N-P T cell aging: a review of the transcriptional changes determined from genome-wide analysis. Front Immunol (2013) 4:121 10.3389/fimmu.2013.00121 23730304 PMC3657702

[35] Blackman MA Woodland DL The narrowing of the CD8 T cell repertoire in old age. Curr Opin Immunol (2011) 23:537–42 10.1016/j.coi.2011.05.005 21652194 PMC3163762

[36] Fujisaki J Wu J Carlson AL Silberstein L Putheti P Larocca R In vivo imaging of Treg cells providing immune privilege to the haematopoietic stem-cell niche. Nature (2011) 474:216–9 10.1038/nature10160 21654805 PMC3725645

[37] Martinez-Agosto JA Mikkola HKA Hartenstein V Banerjee U The hematopoietic stem cell and its niche: a comparative view. Genes Dev (2007) 21:3044–60 10.1101/gad.1602607 18056420

[38] Haynes L Maue AC Effects of aging on T cell function. Curr Opin Immunol (2009) 21:414–7 10.1016/j.coi.2009.05.009 19500967 PMC3800142

[39] Almanzar G Schwaiger S Jenewein B Keller M Herndler-Brandstetter D Würzner R Long-term cytomegalovirus infection leads to significant changes in the composition of the CD8+ T-cell repertoire, which may be the basis for an imbalance in the cytokine production profile in elderly persons. J Virol (2005) 79:3675–83 10.1128/JVI.79.6.3675-3683.2005 15731261 PMC1075718

[40] Yang H Youm Y-H Vandanmagsar B Ravussin A Gimble JM Greenway F Obesity increases the production of proinflammatory mediators from adipose tissue T cells and compromises TCR repertoire diversity: implications for systemic inflammation and insulin resistance. J Immunol (2010) 185:1836–45 10.4049/jimmunol.1000021 20581149 PMC4829921

[41] Johnson PLF Yates AJ Goronzy JJ Antia R Peripheral selection rather than thymic involution explains sudden contraction in naive CD4 T-cell diversity with age. Proc Natl Acad Sci U S A (2012) 109:21432–7 10.1073/pnas.1209283110 23236163 PMC3535632

